# Scoring Systems to Evaluate the Mortality Risk of Patients with Emphysematous Cystitis: A Retrospective Observational Study

**DOI:** 10.3390/jpm13020318

**Published:** 2023-02-13

**Authors:** Yi-Hsuan Chen, Ming-Shun Hsieh, Sung-Yuan Hu, Shih-Che Huang, Che-An Tsai, Yi-Chun Tsai

**Affiliations:** 1 Department of Emergency Medicine, Taichung Veterans General Hospital, Taichung 40705, Taiwan; 2 Department of Post-Baccalaureate Medicine, College of Medicine, National Chung Hsing University, Taichung 402202, Taiwan; 3 Department of Emergency Medicine, Taipei Veterans General Hospital, Taoyuan Branch, Taoyuan 330, Taiwan; 4 Department of Emergency Medicine, Taipei Veterans General Hospital, Taipei 11217, Taiwan; 5 School of Medicine, National Yang Ming Chiao Tung University, Taipei 11221, Taiwan; 6 Institute of Medicine, Chung Shan Medical University, Taichung 40201, Taiwan; 7 Department of Emergency Medicine, Chung Shan Medical University Hospital, Taichung 40201, Taiwan; 8 Lung Cancer Diagnosis and Treatment Research Center, Chung Shan Medical University Hospital, Taichung 40201, Taiwan; 9 Division of Infectious Disease, Department of Internal Medicine, Taichung Veterans General Hospital, Taichung 40705, Taiwan

**Keywords:** emphysematous cystitis (EC), Mortality in Emergency Department Sepsis (MEDS) score, National Early Warning Score (NEWS), scoring systems, receiver operating characteristic curve (ROC)

## Abstract

Background: Emphysematous cystitis (EC) is a complicated urinary tract infection (UTI) characterized by gas formation within the bladder wall and lumen. Immunocompetent people are less likely to suffer from complicated UTIs, but EC usually occurs in women with poorly controlled diabetes mellitus (DM). Other risk factors of EC include recurrent UTI, neurogenic bladder disorder, blood supply disorders, and prolonged catheterization, but DM is still the most important of all aspects. Our study investigated clinical scores in predicting clinical outcomes of patients with EC. Our analysis is unique in predicting EC clinical outcomes by using scoring system performance. Materials and Methods: We retrospectively collected EC patient data from the electronic clinical database of Taichung Veterans General Hospital between January 2007 and December 2020. Urinary cultures and computerized tomography confirmed EC. In addition, we investigated the demographics, clinical characteristics, and laboratory data for analysis. Finally, we used a variety of clinical scoring systems as a predictor of clinical outcomes. Results: A total of 35 patients had confirmed EC, including 11 males (31.4%) and 24 females (68.6%), with a mean age of 69.1 ± 11.4 years. Their hospital stay averaged 19.9 ± 15.5 days. The in-hospital mortality rate was 22.9%. The Mortality in Emergency Department Sepsis (MEDS) score was 5.4 ± 4.7 for survivors and 11.8 ± 5.3 for non-survivors (*p* = 0.005). For mortality risk prediction, the AUC of ROC was 0.819 for MEDS and 0.685 for Rapid Emergency Medicine Score (REMS). The hazard ratio of univariate and multivariate logistic regression analyses of REMS for EC patients was1.457 (*p* = 0.011) and 1.374 (*p* = 0.025), respectively. Conclusion: Physicians must pay attention to high-risk patients according to clinical clues and arrange imaging studies as soon as possible to confirm the diagnosis of EC. MEDS and REMS are helpful for clinical staff in predicting the clinical outcome of EC patients. If EC patients feature higher scores of MEDS (≥12) and REMS (≥10), they will have higher mortality.

## 1. Introduction

Emphysematous cystitis (EC) is a less frequent form of complicated lower urinary tract infection (UTI) and is a potentially life-threatening condition characterized by gas within the bladder wall and lumen as a result of fermentation from bacterial overgrowth. The earliest mention of a case of pneumaturia appeared in 1671, and the gas within the bladder wall was reported in the late 1800s. The correlation between pneumaturia and EC was later characterized in 1961 [1,2,3,4]. 

EC has a highly variable presentation and course ranging from asymptomatic to severe sepsis. However, delayed diagnosis can lead to severe necrotizing infections and become life-threatening [3,4,5]. The overall mortality rate of EC patients is 7–14% [2,3]. Imaging studies are necessary tools to detect the gas in EC patients, and computed tomography (CT) is the gold standard to make sure of the diagnosis of EC. Some predictive scoring models have been established and are available to quickly stratify patients and identify potentially critical conditions in the emergency department (ED) [6,7,8,9,10,11,12,13]. However, there are no established scoring systems for mortality risk prediction of EC in reviewing the literature. We gathered clinical scores of EC patients and analyzed their demographics and laboratory findings concerning their clinical outcomes. We here aimed to validate the performance of various clinical scoring systems to assess this disease’s severity and clinical outcomes. We applied these clinical scoring systems to evaluate the mortality risk.

## 2. Materials and Methods

### 2.1. Data Collection and Definition 

The institutional review board of Taichung Veterans General Hospital (TCVGH), Taichung, Taiwan, approved our study (CE21215A). It was a single-hospital-based retrospective observational study on patients with EC. Cases of confirmed EC were each based on the results from at least one culture of urine, blood, pus, and/or tissue and abdominal CT scan in the ED. Patient data were extracted from the electronic medical records of TCVGH, covering a period from January 2007 to December 2020. In addition, we collected the demographics, laboratory investigations, and clinical outcomes. We used the categories of comorbidities due to a few cases in our study, including genitourinary (GU) disease (stones of the urinary tract, benign prostatic hyperplasia, and gynecologic disorders), immune disorders (systemic lupus erythematosus, rheumatoid arthritis, etc.), and tumor (solid and hematologic malignancies). Vital signs were recorded on the arrival of ED. In addition, laboratory data were collected in the ED. The presence of >10^5^ colony-forming units/mL defined a positive urine culture. In-hospital mortality was the primary outcome. We applied a variety of clinical scoring systems to predict clinical outcomes. In addition, we used univariate and multivariate analyses to evaluate the mortality risk.

### 2.2. Scoring Systems

We analyzed the following published clinical scoring systems for the clinical outcome and mortality risk, including the Mortality in Emergency Department Sepsis (MEDS) score, Rapid Emergency Medicine Score (REMS), National Early Warning Score (NEWS), Modified Early Warning Score (MEWS), Rapid Acute Physiology Score (RAPS), and quick Sequential Organ Failure Assessment (qSOFA).

### 2.3. Statistical Analysis

Continuous data are presented as mean ± standard deviation (SD). Categorical data are presented as numbers and percentages. Chi-squared tests were applied to compare categorical data. Mann–Whitney–Wilcoxon U-tests were applied to compare continuous data regarding mortality risks in survivors and non-survivors. To assess possible predictors for mortality, we conducted univariate and multivariate analyses using the Cox regression model, and results were expressed as confidence interval and hazard ratio. We used the area under the curve (AUC) receiver operating of the characteristic curve (ROC) to compare predictive power across different scoring systems. We used cut-off points of scores to stratify mortality risks in terms of sensitivity, specificity, negative predictive value (NPV), and positive predictive value (PPV). A *p*-value < 0.05 was considered statistically significant. Analyses were performed on the Statistical Package for the Social Science (IBM SPSS version 22.0; International Business Machines Corp., New York, NY, USA) and R (Version 4.1.3, R Foundation for Statistical Computing, Vienna, Austria).

## 3. Results

### 3.1. Demographics and Clinical Characteristics 

We summarized the demographics and clinical findings of 35 patients in Table 1, including 11 males (31.4%) and 24 females (68.6%), with their mean age at 69.1 ± 11.4 years. We divided the major clinical syndromes into five categories: fever, abdominal pain, gastrointestinal (GI) symptoms, neurological symptoms, and lower urinary tract syndrome (LUTS). Fever was the leading symptom (34.3%), followed by abdominal pain (22.9%) and GI symptoms (20.0%). Among all those symptoms, the mortality rate of the patients who presented with LUTS was significantly higher (37.5% vs. 3.7%, *p* = 0.030) and of those who presented with GI symptoms (50.0% vs. 11.1%, *p* = 0.033). Diabetes mellitus (DM) of 20 (57.1%) patients was the leading comorbidity. The following disorders were cardiovascular disease (CVD) (*n* = 19, 54.9%), GI disease (*n* = 17, 48.6%), chronic kidney disease (CKD) (*n* = 16, 45.7%), genitourinary disease (*n* = 10, 28.6%), malignancy (*n* = 10, 28.6%), immune disease (*n* = 8, 22.9%), hyperlipidemia (*n* = 7, 20.0%), cerebrovascular accident (CVA) (*n* = 4, 11.4%), gout (*n* = 3, 8.6%), chronic pulmonary obstruction disease (*n* = 3, 8.6%), peripheral arterial occlusive disease (*n* = 2, 5.7%), and transplant (*n* = 1, 2.9%). The rate of malignancy was significantly higher in non-survivors (62.5%) than in survivors (18.5%) (*p* = 0.027). On the other hand, a substantially lower rate of hyperlipidemia was found in non-survivors (0%) than in survivors (51.9%) (*p* = 0.012).

### 3.2. Laboratory Data and Scoring Systems

Laboratory data and scoring systems were summarized in Table 2. Levels of blood urea nitrogen (BUN) (62.4 ± 36.4 vs. 29.0 ± 19.4, *p* = 0.005) and creatinine (Cr) (2.70 ± 1.61 vs. 1.54 ± 1.01, *p* = 0.034) were significantly higher in non-survivors. Levels of bicarbonate (HCO_3_^−^) (18.21 ± 4.72 vs. 22.95 ± 4.46, *p* = 0.033) were significantly lower in non-survivors. The non-survivors had substantially higher scores of MEDS (11.8 ± 5.3 vs. 5.4 ± 4.7, *p* = 0.005) (Table 3).

### 3.3. Microbiology

Bacterial cultures of blood, urine, pus, and/or tissue from individual patients were performed at least once. However, the samples were not collected from all patients; no pus or tissue was collected from patients with conservative treatment and no urine from patients under regular hemodialysis, so the numbers of patients who were assessed for bacterial cultures differed between survival and non-survival groups. Thirty-three patients received urinary and blood cultures with a positive rate of 82.9% (*n* = 29) and 48.6% (*n* = 17), respectively. Only 11 patients provided samples for pus and tissue cultures, and the positive rate was 81.8% (*n* = 9). The leading microorganism was *Escherichia coli* in urine (*n* = 17) and blood (*n* = 8) cultures. Other microorganisms included *Klebsiella pneumoniae*, *Pseudomonas aeruginosa*, *Staphylococcus aureus*, *Enterobacter cloacae*, *Streptococcus pneumoniae*, *Enterococcus faecalis*, and *Candida albicans*.

### 3.4. Clinical Management and Outcomes

The clinical management for EC included antibiotics only, drainage, and surgical intervention. The clinical management showed no significant differences between survivors and non-survivors. Unfortunately, eight patients died in our study, equivalent to a mortality rate of 22.9%.

### 3.5. Univariate and Multivariate Analysis of Risk Factors

We conducted univariate analyses to predict mortality risk on clinical outcomes in these patients. Results were summarized in Table 4. We found higher hazard ratios (HR) in non-survivors for the following: age (HR = 1.134, *p* = 0.041), lactate (HR = 1.019, *p* = 0.030), pH (HR = 0.000, *p* = 0.020), REMS (HR = 1.457, *p*= 0.011), O_2_ use (HR = 4.237, *p* = 0.049), GI symptoms (HR = 6.261, *p* = 0.017), and LUTS (HR = 5.195, *p* = 0.035). In addition, we used multivariate logistic regression analyses for predisposing factors to evaluate the clinical outcomes of these patients. Results showed a higher HR in non-survivors regarding scores of REMS (*p* = 0.025), lactate (*p* = 0.015), and pH (*p* = 0.042) (Table 5).

### 3.6. Receiver Operating Characteristic Curve (ROC)

**Figure 1:**
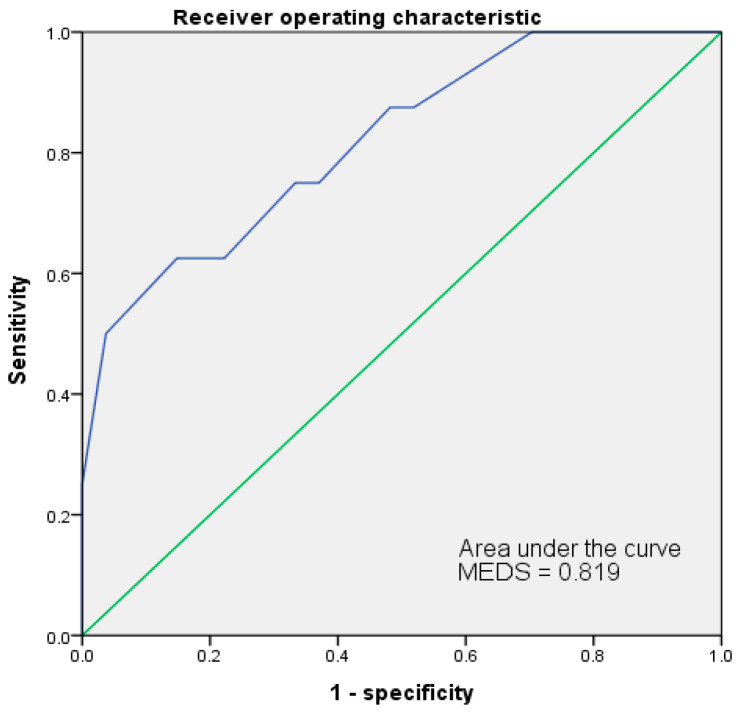
The AUC of ROC for MEDS indicated 0.819 at 12 of the cut-off point to predict the mortality risks of patients with EC. AUC = area under the curve; ROC = receiver operating characteristic curve.

**Figure 2:**
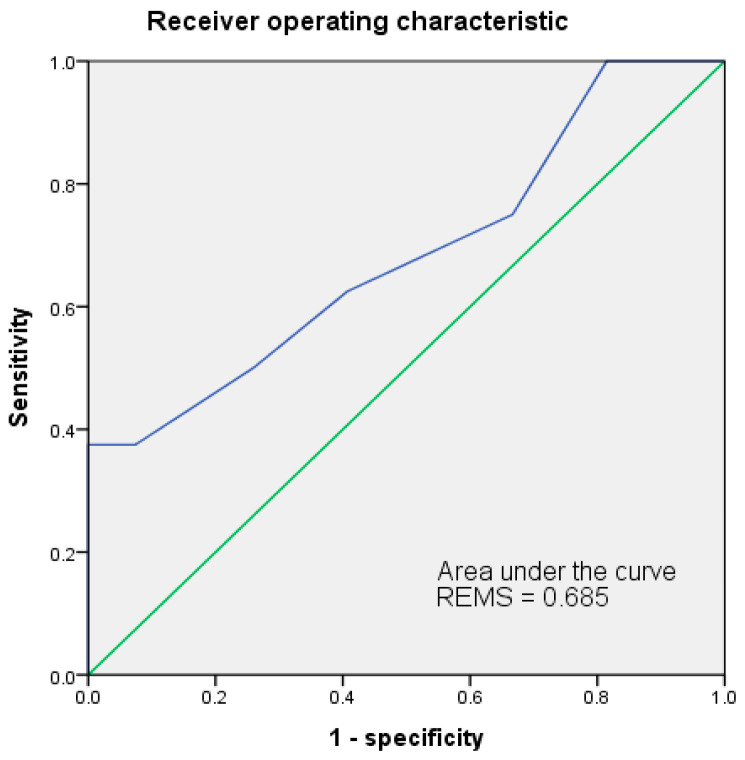
The AUC of ROC for REMS indicated 0.685 at 10 of the cut-off point to predict the mortality risks of patients with EC. AUC = area under the curve; ROC = receiver operating characteristic curve.

The ROC of both MEDS and REMS had been analyzed for accuracy in predicting mortality risks. Results were shown in Figure 1 and Figure 2. The cut-off point of MEDS was 12. The AUC of ROC measured up to 0.819 and had a sensitivity of 62.5% and a specificity of 85.2%. The cut-off point of REMS was 10, and the AUC of ROC reached up to 0.685, had a sensitivity of 37.5% and a specificity of 100.0% (Table 6).

### 3.7. Cumulative Survival Rates by Kaplan–Meier and Discrimination Plots

**Figure 3:**
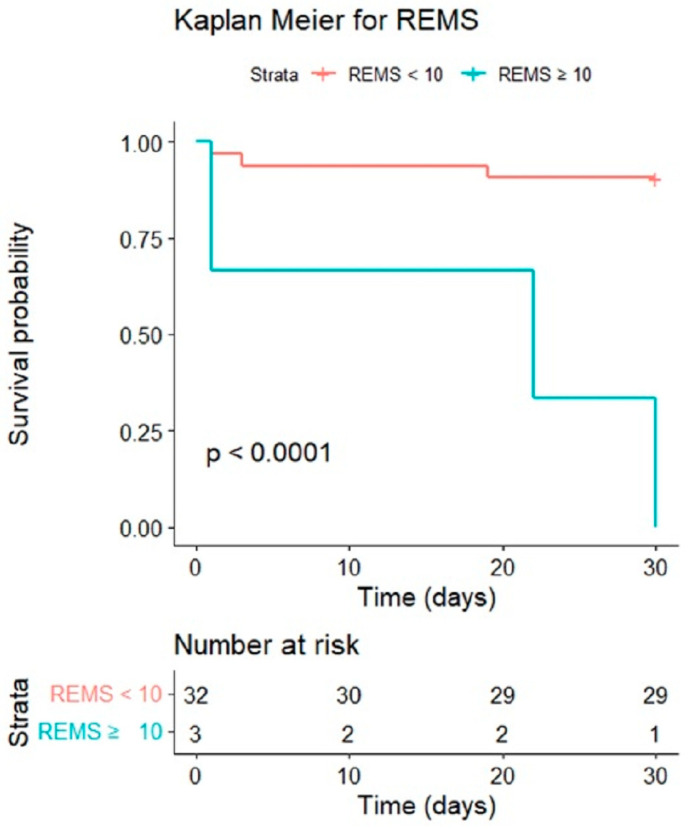
The cumulative survival rates of patients with EC were calculated to predict the 30-day mortality rate by Kaplan–Meier. The cut-off point of REMS was 10.

**Figure 4:**
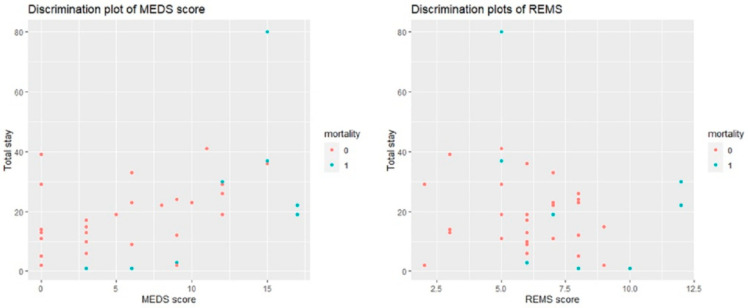
The MEDS was more than 12, and the mortality case numbers were 5, with a mortality rate of 14.3%. The REMS was more than 10, and the mortality case numbers were 3, with a mortality rate of 8.6%.

We analyzed the cumulative survival rates of patients with EC to calculate the 30-day mortality rate by Kaplan–Meier. The cut-off points of REMS (10) demonstrated significant differences between survivors and non-survivors (*p* < 0.001); see Figure 3. The discrimination plots of MEDS and REMS are shown in Figure 4. The MEDS was more than 12, and the mortality case numbers were five, with a mortality rate of 14.3%. The REMS was more than 10, and the mortality case numbers were three, with a mortality rate of 8.6%; see Figure 4.

## 4. Discussion

The mean age of the EC patient group within our study was 69.1 ± 11.4 years higher than in the study of Schicho et al., who reported a mean age of 67.9 ± 14.2 years in 136 EC patients between 2007 and 2016 [14]. Several studies had reported the mortality rate of emphysematous cystitis as about 7–14%. However, the mortality rate was 22.9%, with a mean age of 77.3 ± 6.6 years in our study. Age older than 60 accounted for three-quarters of EC patients, and females were the predominant gender with an incidence of 63.7–65.5% [2,3]. Therefore, we considered older age to contribute to the higher mortality rate [2,3,15,16,17]. In addition, risk factors in EC have been thoroughly investigated, including DM, recurrent UTI, neurogenic bladder (NB), bladder outlet obstruction, blood supply disorders, and prolonged catheterization [2,14,15,16]. According to two extensive case reports, DM was the most common underlying disorder accounting for 60.2–66.7% of EC patients. *Escherichia coli* (58.0–65.6%) and *Klebsiella pneumoniae* (21.0–22.6%) were the leading microorganisms in EC patients [2,3].

The predisposing factors of EC patients, including age, DM, HCVD, CVA, CKD, and NB, were significantly associated with an occurrence of EC [18,19]. However, in univariate analysis, age, lactate, pH, REMS, GI symptoms, and LUTS were significant associated factors in our study. Reviewing the published articles, they never addressed the risk factors associated with the mortality of EC patients, so we focused on risk factors of mortality related to EC. In our study, those older EC patients had LUTS, GI symptoms, neoplasms, high levels of BUN and creatinine, and higher scores of MEDS (≥12) with a higher mortality rate, except for hyperlipidemia. Morin et al. reported high levels of high-density lipoprotein cholesterol (HDL-C) associated with a lower sepsis mortality rate in an animal study. We could not demonstrate HDL-C levels because lipids were not systematically assessed in the ED. We found that a history of hyperlipidemia may be a protective factor in EC patients [20]. The clinical presentations with increased demand for oxygen, tissue hypoperfusion, and impaired renal function in patients with infectious process were often correlated with sepsis, thus increasing the mortality rate. Our report disclosed a higher incidence of high BUN and creatinine levels in the non-survivors than in the survivors of EC patients [21,22]. GI symptoms accounted for 75% of EC patients. However, the relationship and mechanism of association between GI symptoms and mortality of EC patients should be further investigated [23,24]. Patients with underlying malignancy and the presentation of LUTS were associated with a higher mortality rate. We speculated that the diagnosis of EC could be challenging to identify in those immunocompromised patients who used prednisolone, cyclosporine, actarit, etc., with atypical symptoms of UTI [2,25,26,27]. Thus, the diagnosis might have been delayed and the condition could become life-threatening, resulting in a higher mortality rate [28].

Several clinical scoring systems can quickly stratify patients and identify potentially critical conditions in the ED and intensive care units based on variable physiological parameters. Using those functional and easily employed clinical scoring systems, physicians can decide on the patient’s treatment options on short notice and start appropriate antibiotics treatment, drainage, and/or surgical intervention [29,30,31,32,33]. The MEDS score identifies significant correlates of mortality and allows the stratification of patients according to the mortality risk. It is also widely used to predict the mortality risk for patients with community-acquired bacteremia in Taiwan. In a recent study, the REMS score was applied to patients with COVID-19 and influenced its risk stratification [34,35,36,37,38]. Olsson et al. created the REMS score in 2004 and the parameters were listed in Table 7. The REMS score was a powerful predictor of in-hospital mortality in non-surgical ED patients [39].

In this single-center retrospective study, we found higher scores of MEDS and REMS in the non-survivors with the univariate analysis. Multivariate logistic regression demonstrated the AUC of ROC of MEDS amounting to 0.819 as a tool in predicting the mortality risk of EC patients with a cut-off point of 12. The MEDS score was first developed by Shapiro et al. in 2003. It was based on clinical parameters, including lower respiratory infection, respiratory difficulty, septic shock, altered mental status, platelet count, band proportion, age, terminal disease, and nursing home residence (Table 8) to predict mortality accurately in ED patients with suspected infections. The mortality rate was 1.1% on a score of 0–4, 4.4% on a score of 5–7, 9.3% on a score of 8–12, 16.1% on a score of 13–15, and 39% on a score of >15 [40]. The mortality rate was 14.3% if MEDS ≥ 12 in our study. The findings supported the good discrimination of MEDS in predicting the mortality of EC patients. Our analysis uniquely indicated EC clinical outcomes through scoring system performance.

## 5. Limitations

First, this was a single-center study with retrospective nature. Second, it was a study of small sample size, and due to the extended study duration, we enrolled less than three patients per year on average. Third, clinical symptoms were investigated retrospectively without uniform criteria, resulting in inevitable bias. Fourth, data on EC characteristics might only be partially represented, such as the status of glucose control (HbA1C), to evaluate their correlation with mortality.

## 6. Conclusions

Physicians must pay attention to high-risk patients according to clinical clues and arrange imaging studies as soon as possible to confirm the diagnosis of EC. MEDS and REMS are helpful for clinical staff to predict the clinical outcome in EC patients. If EC patients feature higher scores of MEDS (≥12) and REMS (≥10), they would have higher mortality rates of 14.3% and 8.6%, respectively, in our study. We recommend developing a new scoring system to predict the mortality risk of EC using more significant case numbers of multicentric approaches to perform more powerful analyses in the future.

## Figures and Tables

**Table 1:** Demographics and clinical characteristics of emphysematous cystitis.

General Data	All (*n* = 35)	Survival (*n* = 27)	Expired (*n* = 8)	*p*-Value
Male ^f^	11 (31.4%)	10 (37.0%)	1 (12.5%)	0.387
Age	69.4 ± 11.4	66.7 ± 11.4	77.3 ± 6.6	0.027 *
Vital signs				
SBP	135.6 ± 30.5	135.9 ± 32.0	134.8 ± 27.2	0.665
DBP	83.2 ± 26.2	82.6 ± 24.6	85.1 ± 32.9	0.736
MAP	100.7 ± 25.6	100.4 ± 25.1	101.7 ± 29.2	0.630
HR	98.54 ± 20.51	96.30 ± 19.61	106.13 ± 22.99	0.224
RR	18.8 ± 2.2	18.6 ± 2.1	19.5 ± 2.8	0.240
BT	37.15 ± 1.06	37.32 ± 0.95	36.58 ± 1.29	0.109
GCS	14.5 ± 1.6	14.4 ± 1.8	14.8 ± 0.7	1.000
SpO_2_	97.5 ± 3.6	97.7 ± 2.3	96.6 ± 6.4	0.682
Symptoms				
Fever ^f^	12 (34.3%)	11 (40.8%)	1 (12.5%)	0.216
Flank pain ^f^	8 (22.9%)	8 (29.6%)	0 (0%)	0.154
Abdominal pain ^f^	8 (22.9%)	7 (25.9%)	1 (12.5%)	0.648
Consciousness change ^f^	4 (11.4%)	4 (14.8%)	0 (0%)	0.553
GI symptoms ^f^	7 (20.0%)	3 (11.1%)	4 (50.0%)	0.033 *
LUTS ^f^	4 (11.4%)	1 (3.7%)	3 (37.5%)	0.030 *
Nonspecific ^f^	8 (22.9%)	7 (25.9%)	1 (12.5%)	0.648
Comorbidities				
Cardiovascular disease ^f^	19 (54.9%)	17 (63.0%)	2 (25.0%)	0.105
DM ^f^	20 (57.1%)	17 (63.0%)	3 (37.5%)	0.246
Hyperlipidemia ^f^	14 (20.0%)	14 (51.9%)	0 (0%)	0.012 *
Gout ^f^	3 (8.6%)	2 (7.4%)	1 (12.5%)	0.553
CVA ^f^	4 (11.4%)	3 (11.1%)	1 (12.5%)	1.000
COPD ^f^	3 (8.6%)	3 (11.1%)	0 (0%)	1.000
GI disease ^f^	17 (48.6%)	11 (40.7%)	6 (75.0%)	0.121
Chronic renal failure ^f^	16 (45.7%)	12 (44.4%)	4 (50.0%)	1.000
Transplant ^f^	1 (2.9%)	1 (3.7%)	0 (0%)	0.479
GU disease ^f^	10 (28.6%)	9 (33.3%)	1 (12.5%)	0.390
Immune disorder ^f^	8 (22.9%)	7 (25.9%)	1 (12.5%)	0.648
Tumor ^f^	10 (28.6%)	5 (18.5%)	5 (62.5%)	0.027 *

Chi-squared test. ^f^ Fisher’s Exact test. Mann–Whitney U-test. * *p* < 0.05, statistically significant. Continuous data were expressed as mean ± SD. Categorical data were expressed as number and percentage. Abbreviations: BT, body temperature; COPD, chronic obstructive pulmonary disease; CVA, cerebrovascular accident; DBP, diastolic blood pressure; DM, diabetes mellitus; GCS, Glasgow Coma Scale; GI: gastrointestinal; GU, genitourinary; HR, heart rate; LUTS, lower urinary tract symptoms; MAP, mean blood pressure; RR, respiratory rate; SBP, systolic blood pressure.

**Table 2:** Laboratory data of emphysematous cystitis.

Laboratory Data	All (*n* = 35)	Survival (*n* = 27)	Expired (*n* = 8)	*p*-Value
Blood cell counts				
WBC (×10^3^ counts/mm^3^)	17.10 ± 10.42	18.26 ± 10.30	13.15 ± 10.47	0.283
Hemoglobin (g/dL)	10.43 ± 2.23	10.56 ± 2.35	9.98 ± 1.86	0.368
Platelet (×10^3^ counts/mm^3^)	253.06 ± 163.66	265.19 ± 148.23	212.13 ± 214.49	0.204
Band (%)	3.0 ± 11.6	0.8 ± 1.9	10.4 ± 23.6	0.101
Neutrophil (Segment) (%)	107.22 ± 41.30	103.89 ± 40.87	122.23 ± 43.62	0.130
Biochemistry				
Albumin (g/dL)	2.85 ± 0.70	2.88 ± 0.72	2.73 ± 0.62	0.568
Total bilirubin (mg/dL)	1.01 ± 1.72	1.07 ± 1.97	0.82 ± 0.50	0.657
ALT (U/L)	29.2 ± 30.4	28.8 ± 30.3	30.6 ± 32.6	0.885
BUN (mg/dL)	36.7 ± 27.6	29.0 ± 19.4	62.4 ± 36.4	0.005 **
Cr (mg/dL)	1.81 ± 1.24	1.54 ± 1.01	2.70 ± 1.61	0.034 *
CRP (mg/dL)	17.80 ± 12.12	17.65 ± 12.28	18.35 ± 12.41	0.967
Lactate (mg/dL)	26.26 ± 30.78	18.53 ± 20.53	48.49 ± 44.43	0.081
Glucose (mg/dL)	208.9 ± 121.41	214.0 ± 130.7	191.5 ± 87.9	0.839
PT (s)	11.61 ± 2.20	11.76 ± 2.39	11.16 ± 1.57	0.634
APTT (s)	32.11 ± 8.06	31.83 ± 6.80	32.90 ± 11.50	0.947
Arterial blood gas				
pH	7.40 ± 0.07	7.41 ± 0.06	7.34 ± 0.09	0.094
P_a_CO_2_ (mmHg)	36.58 ± 8.11	37.36 ± 8.33	33.81 ± 7.13	0.276
P_a_O_2_ (mmHg)	69.77 ± 41.22	70.00 ± 39.57	68.96 ± 50.17	0.503
HCO_3_^−^ (mmol)	21.92 ± 4.87	22.95 ± 4.46	18.21 ± 4.72	0.033 *

Chi-squared test. Mann–Whitney U-test. * *p* < 0.05, ** *p* < 0.01, statistically significant. Continuous data were expressed as mean ± SD. Categorical data were expressed as number and percentage. Abbreviations: ALK-P, alkaline phosphatase; ALT, alanine aminotransferase; APTT, activated partial prothrombin time; AST, aspartate aminotransferase; BUN, blood urea nitrogen; CRP, c-reactive protein; Cr, creatinine; PT, prothrombin time.

**Table 3:** Scoring systems to predict the clinical outcomes of emphysematous cystitis.

Scoring Systems	All (*n* = 35)	Survival (*n* = 27)	Expired (*n* = 8)	*p*-Value
MEDS	6.8 ± 5.5	5.4 ± 4.7	11.8 ± 5.3	0.005 **
MEWS	2.7 ± 1.8	2.7 ± 1.8	2.8 ± 1.7	0.900
NEWS	3.4 ± 2.8	2.9 ± 2.5	4.9 ± 3.5	0.146
RAPS	1.9 ± 2.0	1.7 ± 1.9	2.3 ± 2.3	0.585
REMS	6.5 ± 2.4	6.0 ± 2.0	8.1 ± 2.9	0.116
qSOFA	0.3 ± 0.6	0.3 ± 0.65	0.3 ± 0.7	0.550

** *p* < 0.01, statistically significant. Abbreviations: MEDS, Mortality in Emergency Department Sepsis; MEWS, Modified Early Warning Score; NEWS, National Early Warning Score; RAPS, Rapid Acute Physiology Score; REMS, Rapid Emergency Medicine Score; qSOFA, quick Sequential Organ Failure Assessment.

**Table 4:** Hazard ratios and 95% confidence interval of univariate analyses for emphysematous cystitis.

Characteristics	Hazard Ratios	95% Confidence Interval	*p*-Value
Age (years)	1.134	1.005–1.278	0.041 *
Male	0.020	0.000–10.997	0.224
Clinical conditions			
Shock	3.841	0.708–20.857	0.119
Respiratory failure	0.440	0.051–3.769	0.454
ICU admission	0.029	0.000–48.310	0.349
Vital signs			
SBP (mmHg)	1.008	0.983–1.034	0.538
MAP (mmHg)	1.008	0.981–1.036	0.565
HR (bpm)	1.025	0.979–1.073	0.296
RR (bpm)	1.190	0.899–1.575	0.225
BT (°C)	0.378	0.138–1.040	0.060
GCS	1.075	0.550–2.103	0.832
SpO_2_ (%)	0.932	0.805–1.080	0.349
Comorbidities			
Cardiovascular disease	0.348	0.067–1.797	0.208
DM	0.308	0.060–1.589	0.159
CKD	1.667	0.372–7.483	0.504
Hyperlipidemia	0.019	0.000–5.989	0.177
Immune disorder	0.027	0.000–31.769	0.317
Tumor	3.083	0.678–14.023	0.145
Laboratory data			
White blood cell (counts/µL)	1.000	1.000–1.000	0.894
Hemoglobin (g/dL)	1.111	0.782–1.578	0.557
Platelet (×10^3^ counts/µL)	1.000	1.000–1.000	0.510
Albumin (g/dL)	4.761	0.640–35.430	0.128
Total bilirubin (mg/dL)	0.782	0.278–2.198	0.641
ALT (U/L)	1.003	0.981–1.026	0.772
BUN	1.036	1.013–1.060	0.002 **
Cr	1.877	1.120–3.145	0.017 *
C-reactive protein (mg/dL)	0.964	0.892–1.042	0.354
Lactate (mg/dL)	1.019	1.002–1.037	0.030 *
PT (s)	0.843	0.549–1.295	0.435
APTT (s)	0.990	0.897–1.093	0.843
pH	0.000	0.000–0.124	0.020 *
HCO_3_^−^ (mmol/L)	0.990	0.897–1.046	0.843
Scoring systems			
MEDS	1.101	0.940–1.290	0.233
MEWS	1.059	0.704–1.594	0.783
NEWS	1.203	0.954–1.519	0.119
RAPS	1.262	0.885–1.801	0.199
REMS	1.457	1.089–1.950	0.011 *
qSOFA	0.900	0.221–3.660	0.883
Symptoms			
Fever	0.313	0.037–2.613	0.283
Flank pain	0.036	0.000–312.580	0.472
Abdominal pain	0.711	0.082–6.128	0.756
Consciousness change	0.036	0.000–205.275	0.452
GI symptoms	6.261	1.386–28.286	0.017 *
LUTS	5.195	1.126–23.969	0.035 *
Nonspecific	0.033	0.000–63.335	0.376

* *p* < 0.05, ** *p* < 0.01, statistically significant. Abbreviations: ALT, alanine aminotransferase; APTT, activated partial prothrombin time; AST, aspartate aminotransferase; BT, body temperature; BUN, blood urea nitrogen; CKD, chronic kidney disease; Cr, creatinine; DM, diabetes mellitus; GCS, Glasgow Coma Scale; GI, gastrointestinal; HR, heart rate; ICU, intensive care unit; LUTS, lower urinary tract syndromes; MAP, mean arterial pressure; PT, prothrombin time; RR, respiratory rate; SBP, systolic blood pressure. GAPS, Glasgow Admission Prediction Score; MEDS, Mortality in Emergency Department Sepsis; MEWS, Modified Early Warning Score; NEWS, National Early Warning Score; RAPS, Rapid Acute Physiology Score; REMS, Rapid Emergency Medicine Score; qSOFA, quick Sequential Organ Failure Assessment; WPS, Worthing Physiological Scoring system.

**Table 5:** Hazard ratios and 95% confidence interval of univariate and multivariate logistic regression analysis for emphysematous cystitis.

	Univariate			Multivariate		
Variables	HR	95% CI	*p*-Value	HR	95% CI	*p*-Value
REMS	1.457	1.089–1.950	0.011 *	1.374	1.040–1.814	0.025 *
Lactate	1.019	1.002–1.037	0.030 *	1.021	1.004–1.039	0.015 *
pH	<0.0001	0.000–0.124	0.020 *	<0.0001	0.000–0.661	0.042 *

* *p* < 0.05, statistically significant. Multivariate logistic regression analysis adjusted by ICU admission. Abbreviations: CI, confidence interval; CRP, c-reactive protein; HR, Hazard Ratios; MEDS, Mortality in Emergency Department Sepsis Score; NEWS, National Early Warning Score.

**Table 6:** The AUC of ROC, cut-off point, sensitivity specificity, positive predictive value (PPV), negative predictive value (NPV), accuracy, and standard error (SE) of MEDS and NEWS to predict mortality.

Scores	AUC	COP	Sensitivity	Specificity	PPV	NPV	Accuracy	SE	*p* Value
MEDS	0.819	12	62.5%	85.2%	55.6%	88.5%	80.0%	0.087	0.007 **
REMS	0.685	10	37.5%	100.0%	100.0%	84.4%	85.7%	0.117	0.016 *

* *p* < 0.05, ** *p* < 0.01, statistically significant. Abbreviations: AUC, area under the curve; COP, cut-off point; MEDS, Mortality in Emergency Department Sepsis; NPV, negative predictive value; PPV, positive predictive value; REMS, Rapid Emergency Medicine Score; ROC, receiver operating characteristic curve; SE, standard error.

**Table 7:** Variables and pointsof the Rapid Emergency Medicine Score (REMS).

	Points						
Variables	0	+1	+2	+3	+4	+5	+6
Age (years)	<45		45–54	55–64		65–74	>74
Mean arterial pressure	70–109		110–129 50–69	130–159	>159 ≤49		
Heart rate	70–109		110–139 55–69	140–179 40–54	>179 ≤39		
Respiratory rate	12–24	25–34 10–11	6–9	35–49	>49 ≤5		
O_2_ saturation	>89	86–89		75–85	<75		
Glasgow Coma Scale	14 or 15	11–13	8–10	5–7	3 or 4		

**Table 8:** Variables and points of the Mortality in Emergency Department Sepsis (MEDS) score.

Variables	Points
1. Terminal illness with possible death in 1 month	6
2. Hypoxia or tachypnea	3
3. Shock from sepsis	3
4. Platelet count below 150,000	3
5. Granulocytic bands > 5% of white blood cells	3
6. Patient older than 65 years old	3
7. Lower respiratory infection	2
8. Patient is from a nursing home	2
9. Mental status is altered	2

## Data Availability

Readers can access the data and material supporting the study’s conclusions by contacting Sung-Yuan Hu at song9168@pie.com.tw.
